# Efficacy and safety of transarterial chemoembolization plus lenvatinib combined with PD-1 inhibitors versus transarterial chemoembolization plus lenvatinib for unresectable hepatocellular carcinoma: a meta-analysis

**DOI:** 10.3389/fimmu.2024.1466113

**Published:** 2024-08-30

**Authors:** Yue Chen, Luyao Jia, Yu Li, Wenhao Cui, Jukun Wang, Chao Zhang, Chunjing Bian, Tao Luo

**Affiliations:** ^1^ Department of General Surgery, Xuanwu Hospital, Capital Medical University, Beijing, China; ^2^ Emergency Medicine Department, Xuanwu Hospital, Capital Medical University, Beijing, China

**Keywords:** transarterial chemoembolization, lenvatinib, programmed cell death protein-1 inhibitors, unresectable, hepatocellular carcinoma

## Abstract

**Background:**

Locoregional treatment combined with systemic therapy is expected to play a synergistic anticancer role. We conducted this systemic meta-analysis to examine the efficacy and safety of transarterial chemoembolization (TACE) plus lenvatinib with or without programmed cell death protein-1 (PD-1) inhibitors (TLP group) compared with TACE + lenvatinib (TL group) for unresectable hepatocellular carcinoma (uHCC).

**Methods:**

From the inception date to April 2024, the data from PubMed, EMBASE, the Cochrane Library, Ovid, Web of Science, and Clinical Trials. gov were used for meta-analysis. All clinical outcomes of interest included overall survival (OS), progression-free survival (PFS), objective response rate (ORR), disease control rate (DCR), and adverse events (AEs). The hazard ratio (HR) and risk ratio (RR) with 95% confidence intervals (CI) were used to measure the pooled effect.

**Results:**

This study included 10 retrospective cohort studies, including 1128 patients. The OS (HR=0.51; 95% CI: 0.43–0.60, *P < *0.05), PFS (HR=0.52; 95% CI: 0.45–0.61, *P < *0.05), ORR (RR = 1.58; 95% CI: 1.37–1.83; P < 0.05) and DCR (RR = 1.31; 95% CI: 1.20–1.43; P < 0.05) were significantly higher in TLP group than in the TL group. The incidence of AEs was acceptable. Prognostic factor analysis identified that ECOG PS (1/0), Child-Pugh class (B/A), BCLC stage (C/B) and main portal vein invasion (yes/no) were independent prognostic factors for OS. BCLC stage (C/B) and main portal vein invasion (yes/no) were independent prognostic factors for PFS.

**Conclusion:**

The TLP group had better efficacy for uHCC than that of the TL group, with acceptable safety.

**Systematic review registration:**

PROSPERO, identifier (CRD42023420093).

## Introduction

The morbidity and mortality of hepatocellular carcinoma (HCC) remain high worldwide, among which primary liver cancer is the most common. It ranks fifth in the incidence rate and is the third leading cause of cancer death in the world (1). Because of the strong compensatory ability of liver, most patients are diagnosed with HCC when the disease progresses to the middle and late stage. Patients eventually lost the opportunity of surgery, ablation and liver transplantation, resulting in poor prognosis (2). Therefore, the combined treatment of unresectable HCC (uHCC) has attracted much attention.

In SHARP and REFLECT trials, tyrosine kinase inhibitors (TKIs) sorafenib and lenvatinib were recommended as the first-line treatment drugs for advanced HCC respectively (3, 4). Among them, subsequent trials proved that the effect of lenvatinib was not inferior to sorafenib for HCC (5). However, it is found that systemic therapy alone cannot achieve satisfactory survival time for advanced HCC. According to the treatment strategy of Barcelona Clinic Liver Cancer (BCLC), transitional chemoembolization (TACE) is recognized as the first preferred treatment for uHCC (6). But TACE alone has certain limitations. Subsequent clinical trials have proved that the combination of TACE and lenvatinib is more effective than single therapy, with potential effectiveness and safety (7).

With the emergence of refractory and drug-resistant HCC, blocking immune checkpoint pathway by immune checkpoint inhibitors (ICIs) has been found to be a new cancer treatment strategy (8). At present, the most widely studied ICIs is anti-programmed cell death 1 (PD-1)/programmed cell death protein ligand 1 (PD-L1), which can enhance the function of T cells and exert an anti-tumor activity (9). A recent clinical trial (IMbrave150) showed that compared with sorafenib or lenvatinib, the combination of atilizumab (PD-L1) and bevacizumab (anti-vascular endothelial growth factor, VEGF) was a better first-line choice for advanced HCC, and the overall survival(OS) of HCC was significantly prolonged (10). Since then, the beginning of targeted immunotherapy for HCC has begun, and PD-1 has become the second-line therapy for advanced HCC. Therefore, adding PD-1 on the basis of TACE + lenvatinib may optimize the efficacy of the triple therapy and produce more desirable synergistic effect (11, 12).

In the past two years, the efficacy of TACE + lenvatinib + PD-1 triple therapy has achieved encouraging results in many trials. Therefore, we conducted this systemic meta-analysis to compare the efficacy and safety of TACE + lenvatinib + PD-1 triple therapy and TACE + lenvatinib dual therapy in multiple trials, so as to determine a better treatment plan for uHCC patients.

## Materials and methods

Ethical approval was not required for this study, and the article has been reported in line with the PRISMA (Preferred Reporting Items for systemic Reviews and Meta Analyses) checklist (13). This meta-analysis was registered at PROSPERO (CRD42023420093).

### Literature search strategy

**Figure 1:**
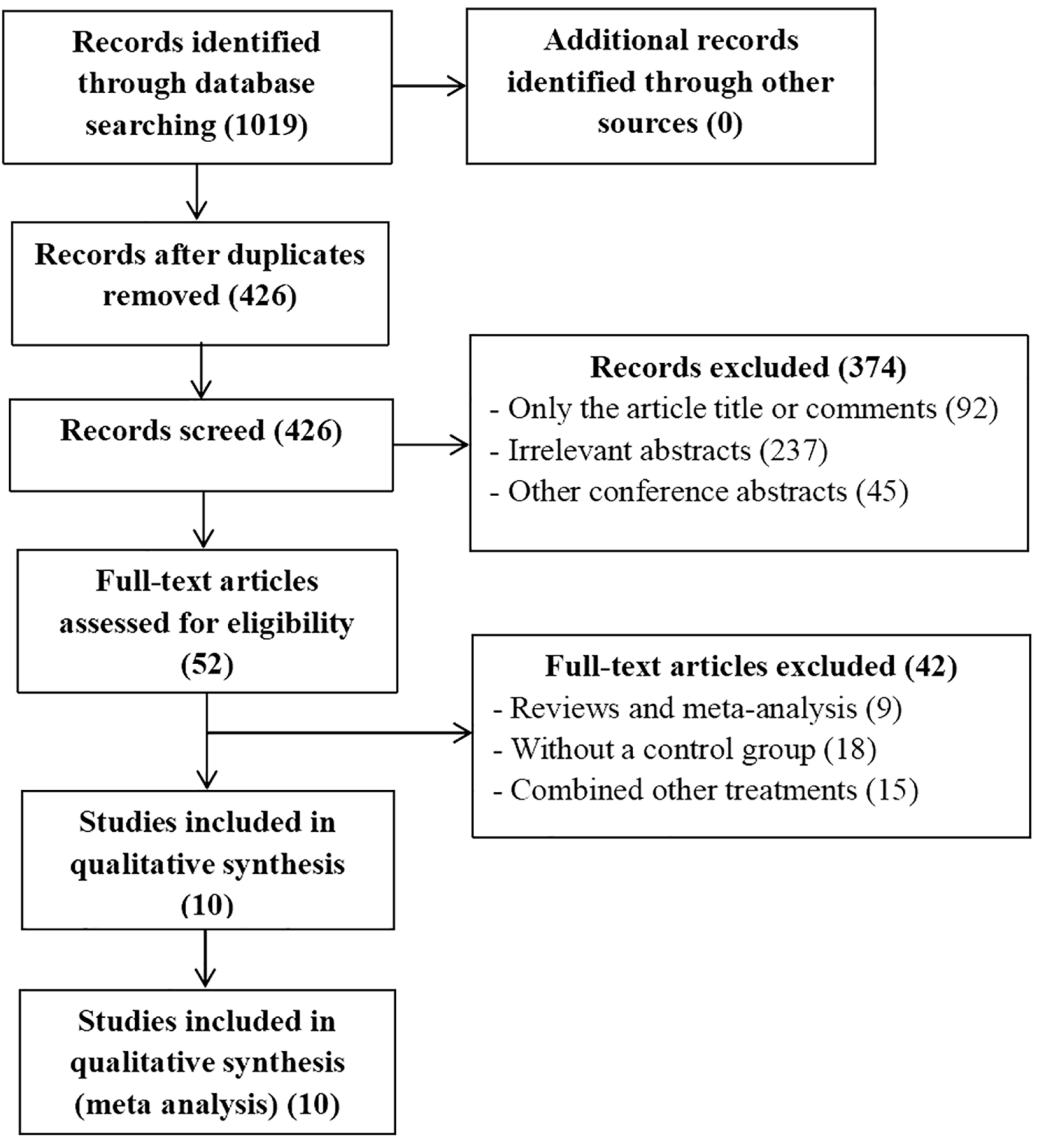
Flowchart of article screening and selection process.

The publication time was limited to when the databases were established until April, 2024. We conducted a systemic search of PubMed, EMBASE, the Cochrane Library, Ovid, Web Of Science, and Clinical Trials.gov databases to identify useful literatures related to this meta-analysis. The MESH terms used in these databases included (“carcinoma, hepatocellular” or “liver cancer” or “HCC” or “liver neoplasm”), (“transarterial chemoembolization” or “TACE”), (“PD-1” or “immunotherapy therapies”) (“Lenvatinib” or “Lenvima”). There are no restrictions on the language of selected literatures. After that, two authors (**XX** and **XX**) independently extracted and confirmed relevant data. The flowchart of the article screening and selection process is presented in the **Figure 1**.

### Study selection

Inclusion criteria: 1) the patients diagnosed with uHCC by imaging and biopsy evidence; 2) the uHCC patients received TACE + lenvatinib + PD-1(TLP) group compared with TACE + lenvatinib (TL) group; 3) the types of study include randomized controlled trials (RCT) and retrospective cohort studies (RCS); 4) the clinical outcomes evaluated were OS, progression-free survival (PFS), objective response rate (ORR), disease control rate (DCR), and adverse events (AEs), which include at least one valuable survival outcome.

Exclusion criteria: 1) the study types included a review, a meta-analysis, a conference abstract, a letter, and a case report; 2) the study lacked effective outcomes data or reported irrelevant outcomes; 3) There is no control group.

### Data extraction and quality assessment

Two reviewers (**XX** and **XX**) independently screened the studies and evaluated the quality of the included studies in a standardized way. Any discrepancy was resolved through a discussion. A third reviewer (**XX**) would decide if necessary. The data extracted from each study include the name of the first author, the year of publication, the design of the study population, the nationality and the clinical characteristics of patients (including sex, age, Child-Pugh class, ECOG PS, BCLC stage). The Newcastle-Ottawa Scale (NOS) was applied for RCS (14). Additionally, tumor responses were determined by the modified response evaluation criteria in solid tumors (mRECIST) or RECIST. The quality assessment of each literature is presented in **Table 1**.

**Table 1:** Baseline characteristics of the studies.

Study	Country	Design	Patients (M/F)		Age (years)		Child-Pugh class (A/B)		ECOG PS (0/1/2)		BLCL (B/C)		PD-1	NOS SCORE
			TACE-L-P	L-P	TACE-L-P	L-P	TACE-L-P	L-P	TACE-L-P	L-P	TACE-L-P	L-P		
Cai 2022 (15)	China	RCS	41 (37/4)	40 (33/7)	51.9 ± 10.3	54.6 ± 11.0	37/4	33/7	33/8/0	28/12/0	/	/	Sintilimab, Tislelizumab, Camrelizumab	8
Chen 2021	China	RCS	70 (37/33)	72 (38/34)	54.2	50	/	/	47/23/0	30/42	47/23	45/27	Pembrolizumab	8
Guo 2022 (16)	China	RCS	75 (65/10)	91 (88/3)	≤60: 66 >60: 25	≤60: 60 >60: 15	13/78	2/73	58/33/0	69/6/0	20/71	20/55	Camrelizumab, Sintilimab	8
Qu 2022 (17)	China	RCS	30 (26/4)	21 (20/1)	55.5 (47.8, 64.3)	50.0 (45.0, 61.0)	28/2	21/0	/	/	1/29	3/18	/	7
Sheng 2024 (18)	China	RCS	113 (95/18)	128 (108/20)	64.48 ± 10.83	62.59 ± 10.58	88/25	99/29	62/36/15	66/42/20	54/59	63/65	Sintilimab, Camrelizumab, Tislelizumab	8
Wang yy 2023 (19)	China	RCS	45 (42/3)	20 (15/5)	54 (18 - 79)	62 (26 - 75)	30/15	18/2	26/19/0	7/13/0	11/34	5/15	Camrelizumab, Sintilimab, Pembrolizumab, Tislelizumab, Nivolumab	7
Wang wj 2023 (20)	China	RCS	54 (49/5)	45 (43/2)	57.0 ± 9.9	60.8 ± 9.4	49/5	42/3	46/8/0	41/4/0	/	/	Sintilimab, Camrelizumab, Toripalimab	8
Wu 2024 (21)	China	RCS	18 (15/3)	23 (18/5)	56.9 ± 8.1	58.1 ± 9.4	18/0	21/2	7/11/0	7/16/0	/	/	Sintilimab, Camrelizumab, Nivolumab, Tislelizumab	8
Xiang 2023 (22)	China	RCS	33 (28/5)	49 (45/4)	51.0 ± 12.2	51.7 ± 11.2	25/8	41/8	22/11	38/11	10/23	22/27	camrelizumab	8
Zou 2023 (23)	China	RCS	70 (59/11)	90 (77/13)	53.6 ± 15.1	52.3 ± 14.8	46/24	61/29	17/53/0	28/62/0	0/70	0/90	Pembrolizumab, Sintilimab	8

M, male; F, female; TACE; transarterial chemoembolization; L, lenvatinib; P, programmed cell death protein-1; ECOG, Eastern Cooperative Oncology Group; BCLC, Barcelona Clinic Liver Cancer; NOS, the Newcastle–Ottawa Scale; RCS, retrospective cohort study; /, not reported.

### Statistical analysis

Hazard ratios (HRs) with 95% confidence intervals (CIs) were calculated to analyze OS and PFS. The risk ratios (RR) with 95% CIs were calculated to analyze ORR, DCR and AEs. A fixed effect model was used for data pooling if no significant heterogeneity among included trials was observed. Otherwise, a random effect model was used. The I^2^ statistic (I^2^ > 50% was deemed to have significant heterogeneity) and χ2 test (P < 0.10 was deemed to suggest significant heterogeneity) were used to assess the heterogeneity among the trials. The funnel plots were performed to detect the existence of publication bias (P < 0.10 was deemed to represent significant publication bias). All analyses were performed using the Revman5.4 software. Statistical test was a two-tailed test, and p < 0.05 was statistically significant.

## Results

### Search results

A total of 10 articles met the inclusion and exclusion criteria, of which 426 were selected after removing duplicates. We excluded 374 articles after reviewing the titles and abstracts of 426 articles. The full text of the remaining 52 articles were evaluated. According to the inclusion and exclusion criteria, 42 studies were excluded. Ultimately, 10 articles including 1128 patients were included in the current meta-analysis, which all studies included were RCS (15–24).

### Study characteristics

The included study characteristics are summarized in **Table 1**. All included articles were published in China from 2022 to 2024. Of the 1128 patients included in our meta-analysis, 848 were male and 280 were female. Furthermore, 549 patients with uHCC received TLP triple therapy, while 579 patients received TL dual therapy. The PD-1 included in all selected articles were mainly Sintilimab, Tislelizumab, Camrelizumab, Pembrolizumab and Nivolumab, which was also depicted in **Table 1**. There were some significant variables which were analyzed in subgroups in the two groups.

### Risk of bias

NOS was used to assess RCS. It contains the selection of subjects, comparability of the groups, and assessment of outcomes, with a maximum of 9 points. Studies with a score of more than 6 were determined to be high quality.

### Meta-analysis outcomes

#### Overall survival and progression-free survival

**Figure 2:**
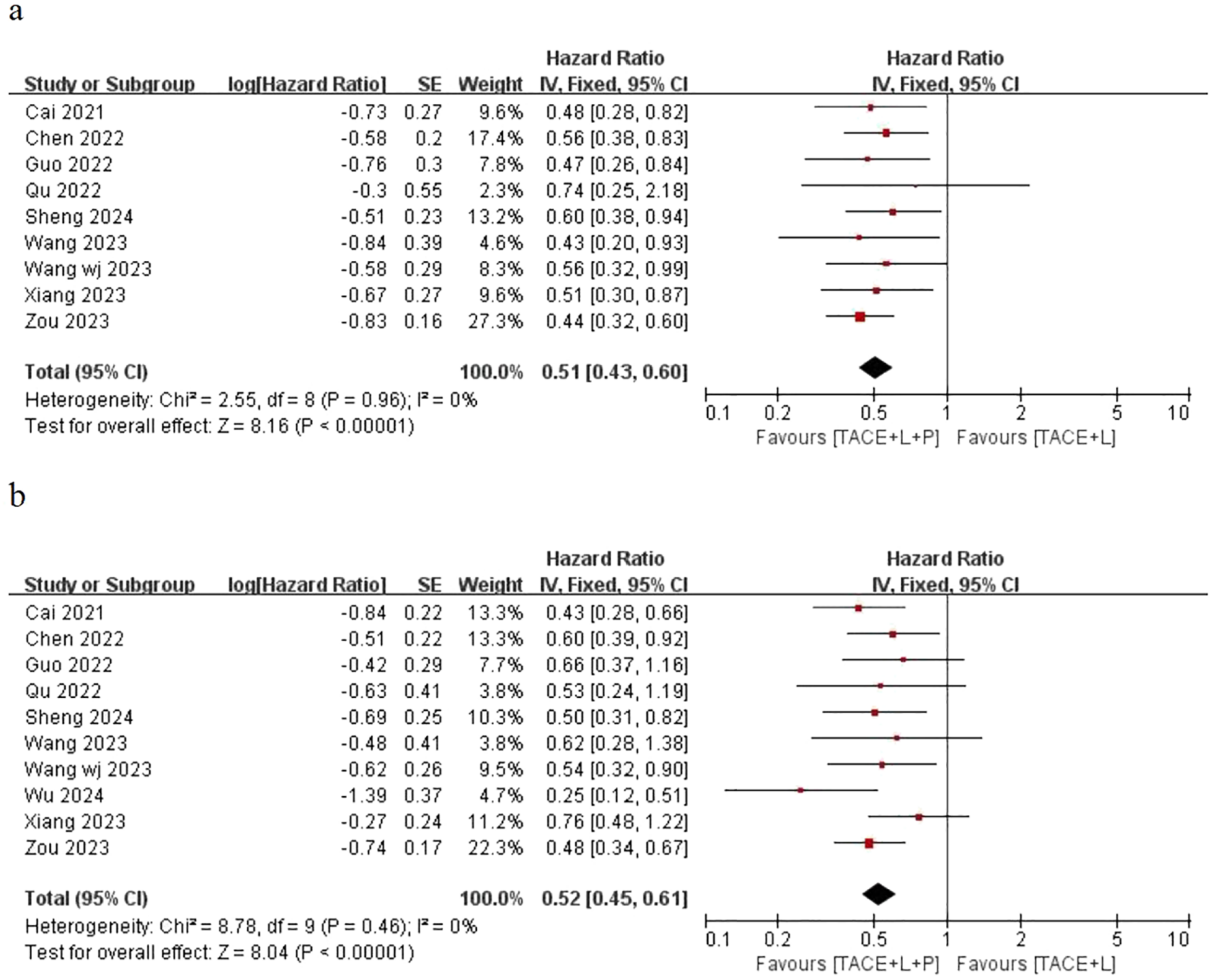
Fixed effect model of OS **(A)** and PFS **(B)** for uHCC with TLP vs TL. OS: overall survival, PFS: progression-free survival, uHCC: unresectable hepatocellular carcinoma, T: transarterial chemoembolization, L: lenvatinib, P: programmed cell death protein-1, CI: confidence intervals.

Only nine articles provided the outcomes of OS and 10 articles provided the outcomes of PFS for the two groups, including the point estimate (HR) and its 95% CI. The meta-analysis indicated that patients with uHCC in the TLP group had significantly longer OS (HR=0.51; 95% CI: 0.43–0.60, *P < *0.05) and PFS (HR=0.52; 95% CI: 0.45–0.61, *P < *0.05) than those in the TL group. The findings indicated that the TLP triple therapy could significantly prolonged survival time (**Figure 2**).

#### Disease control rate and objective response rate

**Figure 3:**
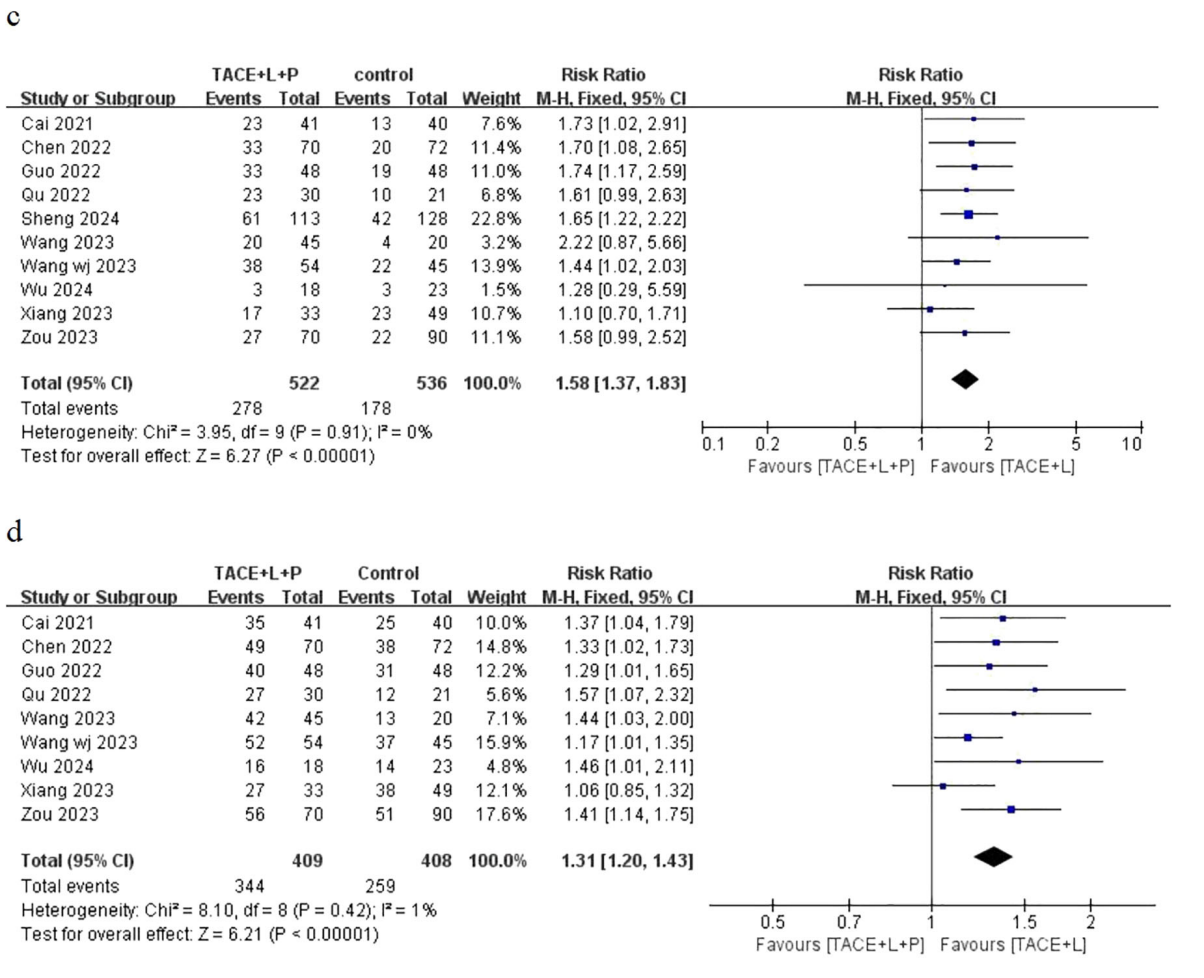
Fixed effect model of ORR **(C)** and DCR **(D)** for uHCC with TLP vs TL. ORR: objective response rate, DCR: disease control rate, uHCC: unresectable hepatocellular carcinoma, T: transarterial chemoembolization, L: lenvatinib, P: programmed cell death protein-1, CI: confidence intervals.

Ten articles have provided the outcomes of ORR and only nine articles have provided the outcomes of DCR for the two groups, including the point estimate (RR) and its 95% CI. The meta-analysis indicated that patients with uHCC in the TLP group had significantly better ORR (RR = 1.58; 95% CI: 1.37–1.83; *P* < 0.05) and DCR (RR = 1.31; 95% CI: 1.20–1.43; *P* < 0.05) than those in the TL group (**Figure 3**). Similarly, the results also showed that the TLP triple therapy was more effective than the TL dual therapy.

#### Prognostic factor analysis for overall survival and progression-free survival

The results based on univariate and multivariate analysis data from included trials in both groups showed that ECOG PS (1/0): (HR=1.18; 95% CI: 1.02-1.36, *P < *0.05), Child-Pugh class (B/A): (HR=1.83; 95% CI: 1.54-2.17, *P < *0.05), BCLC stage (C/B): (HR=1.85; 95% CI: 1.29-2.66, *P < *0.05) and main portal vein invasion (yes/no): (HR=1.25; 95% CI: 1.05-1.50, *P < *0.05) were independent prognostic factors for OS. Similarly, the results showed that BCLC stage (C/B): (HR=1.60; 95% CI: 1.04-2.46, *P < *0.05) and main portal vein invasion (yes/no): (HR=1.27; 95% CI: 1.02-1.59, *P < *0.05) were independent prognostic factors for PFS (**Table 2**).

**Table 2:** Analyses of prognostic factors for OS and PFS of TLP group vs TL group.

Factor	Analysis of OS			Analysis of PFS		
	No.	HR [95% CI]	P	No.	HR [95% CI]	P
AFP((μg/L) ≥400/<400	4	1.23 [0.96, 1.56]	0.1	5	1.05 [0.86, 1.29]	0.62
ECOG PS 1/0	4	1.18 [1.02, 1.36]	**0.03**	5	1.45 [0.96, 2.19]	0.07
Child-Pugh class B/A	5	1.83 [1.54, 2.17]	**<0.001**	4	1.32 [0.81, 2.14]	0.27
BCLC stage C/B	3	1.85 [1.29, 2.66]	**0.001**	2	1.60 [1.04, 2.46]	**0.03**
main portal vein invasion yes/no	4	1.25 [1.05, 1.50]	**0.01**	5	1.27 [1.02, 1.59]	**0.03**
Extrahepatic metastasis yes/no	4	1.42 [0.89, 2.26]	0.15	3	1.44 [0.88, 2.33]	0.14

OS, overall survival; PFS, progression-free survival; T; transarterial chemoembolization; L, lenvatinib; P, programmed cell death protein-1; AFP, alpha fetoprotein; ECOG, Eastern Cooperative Oncology Group; BCLC, Barcelona Clinic Liver Cancer; No., number; HR, hazard ratio; CI, confidence intervals. Bold values indicate significant risk factors affecting OS and PFS for uHCC patients in the TLP group vs. TLP group.

#### Adverse events

Ten included studies reported the incidence of grade 3/4 AEs and only 9 studies reported all grades AEs. The common incidence of grade 3-4 AEs and all grade AEs included abdominal pain, decreased appetite, hypertension, nausea, diarrhea, rash, hand-foot syndrome, elevated aspartate aminotransferase (AST), elevated alanine aminotransferase (ALT), thrombocytopenia, thyroid dysfunction (**Table 3**). The frequency of all grades AEs was similar in both groups. The incidence of 3/4 grade nausea (RR = 4.40; 95% CI: 1.42-13.61; *P* < 0.05), rash (RR = 2.75; 95% CI: 1.13-6.70; *P* < 0.05), hand-foot syndrome (RR = 2.51; 95% CI: 1.29-4.89; *P* < 0.05) and thyroid dysfunction (RR = 6.12; 95% CI: 1.49, 25.19; *P* < 0.05) were more frequent in the TLP group than in the TL group.

**Table 3:** Adverse events of TLP group vs TL group.

Adverse Events	All grades			Grade 3/4		
	No.	RR [95% CI]	P	No.	RR [95% CI]	P
Abdominal pain	7	0.92 [0.73, 1.17]	0.52	7	1.00 [0.47, 2.11]	0.99
Decreased appetite	6	1.22 [0.55, 2.72]	0.63	7	1.33 [0.65, 2.70]	0.44
Hypertension	8	1.23 [0.96, 1.59]	0.1	8	1.74 [1.10, 2.74]	0.51
Nausea	5	0.84 [0.50, 1.40]	0.5	6	4.40 [1.42, 13.61]	**0.01**
Diarrhea	9	1.09 [0.80, 1.48]	0.58	10	1.45 [0.77, 2.71]	0.25
Rash	6	1.39 [0.90, 2.15]	0.14	7	2.75 [1.13, 6.70]	**0.03**
Hand-foot syndrome	5	1.00 [0.70, 1.42]	1	6	2.51 [1.29, 4.89]	**0.007**
Elevated AST	5	1.18 [0.90, 1.56]	0.23	6	1.32 [0.76, 2.31]	0.32
Elevated ALT	5	1.05 [0.83, 1.32]	0.71	6	0.95 [0.47, 1.93]	0.89
Thrombocytopenia	7	1.19 [0.84, 1.70]	0.32	8	1.31 [0.70, 2.46]	0.40
Thyroid dysfunction	7	1.48 [0.89, 2.47]	0.13	7	6.12 [1.49, 25.19]	**0.01**

T, transarterial chemoembolization; L, lenvatinib; P, programmed cell death protein-1; AST, aspartate aminotransferase; ALT, alanine aminotransferase; No., number; RR, relative risk; CI, confidence intervals. The bold values indicate that compared to the TLP group, the TL group of uHCC patients experienced more frequent and severe grade 3-4 adverse events, which should be taken seriously.

#### Publication bias

The funnel plots were applied to show the publication bias of this meta-analysis. The funnel plots of outcomes for OS, PFS, ORR, and DCR are shown in the **Supplementary Materials**. In general, the probability of publication bias is low as the scatter points were distributed symmetrically in the inverted funnel.

## Discussion

With the widespread concern of locoregional treatment combined with systemic targeted immunotherapy in the treatment of uHCC, more experiments have been conducted to study the efficacy of TACE plus lenvatinib combined with PD-1 for advanced HCC. The results of our meta-analysis showed that compared with the TL group, the TLP group achieved longer OS and PFS, better ORR and DCR, with low heterogeneity. Treatment-related AEs were acceptable. Prognostic factor analysis identified ECOG PS (1/0), Child Pugh class (B/A), BCLC stage (C/B), and main portal vein invasion (yes/no) as independent prognostic factors for OS. BCLC stage (C/B) and main portal vein invasion (yes/no) were identified as independent prognostic factors for PFS. As far as we know, our meta-analysis is a relatively comprehensive study at present, providing more reliable evidence for uHCC patients.

Lenvatinib is a multi-targeted receptor TKI that targets VEGFR1-3, as well as fibroblast growth factor receptors (FGFR)1-4 (25). The LAUNCH trial showed TACE + lenvatinib had longer OS and PFS than lenvatinib alone for uHCC (7). Experts agree that hypoxia caused by TACE therapy can up-regulate VEGF. TACE combined with lenvatinib can reverse the high secretion of antigen factors and inhibit the recurrence and metastasis of residual tumors (26, 27).

Currently, our meta-analysis compared TLP triple therapy with TL dual therapy, and the latter had better results in TLP group. The results of pooled analyses showed a low degree of heterogeneity for the outcomes, using the fixed effect models. The results are consistent with previous studies comparing the combination of TLP versus TL. In a real-world study, TACE combined with PD-1 and TKI significantly improved OS, PFS and ORR for Chinese patients with advanced HCC, with acceptable safety (28). Recently, a prospective study has shown that the triple therapy of TACE + lenvatinib + camrelizumab had satisfactory clinical effect and controllable safety, which further confirmed the clinical efficacy of locoregional treatment combined with targeted immunotherapy for uHCC (29). Better outcomes of TACE + lenvatinib + PD-1 are considered to be attributed to the synergistic antitumor activity of the three combination therapies. PD-1 was added to the TACE + lenvatinib combination therapy to enhance the anti-tumor immune effect. On the one hand, the possible mechanism is that lenvatinib can inhibit IFN-γ signal transduction in tumor cells by targeting FGFR, and the combination of PD-1 and lenvatinib can reverse the immunosuppressive state of the tumor microenvironment, thus improving the immune response rate of PD-1 inhibitors (12, 30). On the other hand, TACE can cause ischemic necrosis of tumor tissue and release a large number of tumor-specific antigens, thus enhancing the anti-tumor immune effect of PD-1 inhibitors (31, 32). In addition, TACE has relatively reduced the adverse reactions of HCC patients to systemic targeted immunotherapy and avoided the frequent drug resistance (33).

In a pooled analysis of all the studies we included, multiple factors were found to be risk factors for OS and PFS for uHCC, which was consistent with previous studies (34). For patients with uHCC, BCLC stage C and major portal vein invasion are both risk factors for OS and PFS of TLP group vs TP group. Both ECOG PS 0 and Child Pugh class A are applied to uHCC patients of TLP group vs TP group. Of note, liver function was evaluated with Child Pugh scores after the treatment procedure, which is significant for prognosis (35). Only patients with better liver function have the ability to tolerate subsequent target immunotherapy and fully utilize the advantages of combination therapy (36).

In addition, the treatment-related AEs were controllable and acceptable in both groups. It is worth noting that the results of our meta-analysis showed that the occurrence frequency of grade 3-4 AEs was higher in the TLP group than that in the TL group, which was safe with appropriate symptomatic treatment (37). The reason why the degree of nausea is more severe in the TLP group is that the triple combination therapy has greater toxic effects. Rash, hand-foot syndrome, and thyroid dysfunction are the most common immune-related adverse events (irAEs), mainly related to the therapeutic mechanism of PD-1 blockade of immune checkpoints. Importantly, the combination therapy for uHCC can lead to inevitable adverse reactions, and its long-term safety requires ongoing attention.

Although the results of this meta-analysis are satisfactory, there are still several limitations. Firstly, due to all the included literatures are retrospective studies, it is difficult to avoid recall bias. Secondly, only 10 studies were included, and there were not enough cases to analyze. Thirdly, considering that all published studies are from China, the conclusion is not applicable to western populations. Last but not least, most of the etiology of HCC are mainly related to hepatitis B virus infection in China. The benefits of combined PD-1 treatment may be related to the etiologies of disease in our study. Therefore, independent prospective clinical trials are needed to further verify the evaluation results.

## Conclusion

This meta-analysis demonstrated that the triple therapy of TACE + lenvatinib +PD-1 was superior to the dual therapy of TACE + lenvatinib with respect to OS, PFS, ORR and DCR, with less occurrence of AEs.

## Data Availability

The original contributions presented in the study are included in the article/**Supplementary Material**. Further inquiries can be directed to the corresponding author.
