# Supplementary material for: Efficacy and safety of transarterial chemoembolization plus lenvatinib combined with PD-1 inhibitors versus transarterial chemoembolization plus lenvatinib for unresectable hepatocellular carcinoma: a meta-analysis
Source: Front Immunol. 2024 Aug 30;15:1466113. doi: 10.3389/fimmu.2024.1466113 (PMC11392794; doi:10.3389/fimmu.2024.1466113)
Supplement: Supplementary file 1 [file DataSheet1.docx]

**Efficacy and safety of transarterial chemoembolization plus lenvatinib combined with PD-1 inhibitors versus transarterial chemoembolization plus lenvatinib for unresectable hepatocellular carcinoma: a meta-analysis**

**Running title: Efficacy of the triple therapy for uHCC**

Yue Chen ^[1]^, Luyao Jia ^[1]^, Yu Li ^[1]^, Wenhao Cui ^[2]^, Jukun Wang ^[1]^, Chao Zhang ^[1]^, Chunjing Bian ^[1]^, Tao Luo *^[1]^

[1] Department of General Surgery, Xuanwu Hospital, Capital Medical University, Beijing, China, 100053

[2] Emergency Medicine Department, Xuanwu Hospital, Capital Medical University, Beijing, China, 100053

*: Corresponding to Dr. Tao Luo ([Taoluo35@126.com](mailto:Taoluo35@126.com)), No. 45 Changchun Street, Xicheng District, Beijing, China


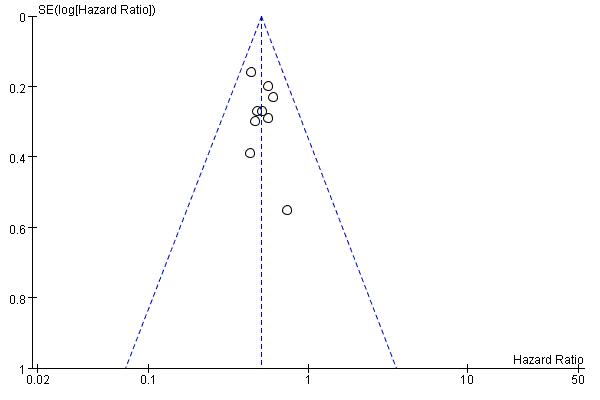


The funnel plots of the publication bias of OS (overall survival)


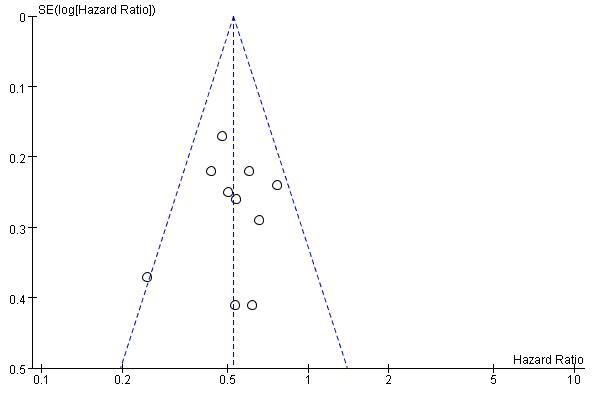


The funnel plots of the publication bias of PFS (progression-free survival)


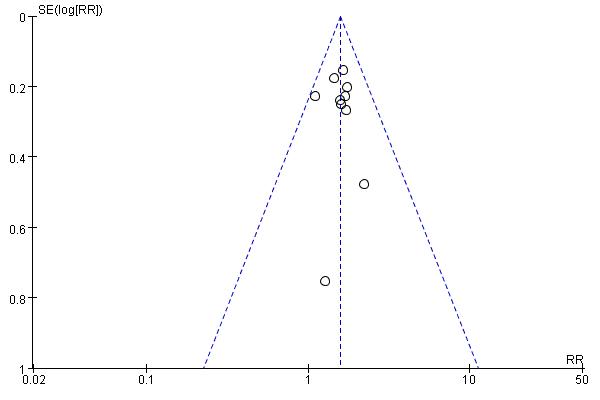


The funnel plots of the publication bias of ORR (objective response rate)


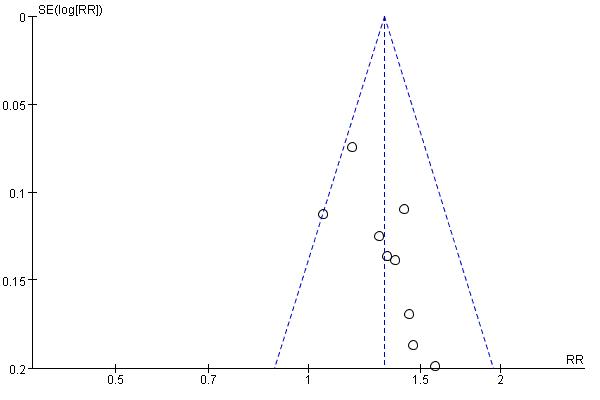


The funnel plots of the publication bias of DCR (disease control rate)
